# Reconstruction of viral population structure from next-generation sequencing data using multicommodity flows

**DOI:** 10.1186/1471-2105-14-S9-S2

**Published:** 2013-06-28

**Authors:** Pavel Skums, Nicholas Mancuso, Alexander Artyomenko, Bassam Tork, Ion Mandoiu, Yury Khudyakov, Alex Zelikovsky

**Affiliations:** 1 Laboratory of Molecular Epidemiology and Bioinformatics, Division of Viral Hepatitis, Centers for Disease Control and Prevention, 1600 Clifton Road NE, 30333 Atlanta, GA, USA; 2 Department of Computer Science, Georgia State University, 34 Peachtree str., 30303, Atlanta, GA, USA; 3 Department of Computer Science and Engineering, University of Connecticut, 06269, Storrs, CT, USA

## Abstract

**Background:**

Highly mutable RNA viruses exist in infected hosts as heterogeneous populations of genetically close variants known as quasispecies. Next-generation sequencing (NGS) allows for analysing a large number of viral sequences from infected patients, presenting a novel opportunity for studying the structure of a viral population and understanding virus evolution, drug resistance and immune escape. Accurate reconstruction of genetic composition of intra-host viral populations involves assembling the NGS short reads into whole-genome sequences and estimating frequencies of individual viral variants. Although a few approaches were developed for this task, accurate reconstruction of quasispecies populations remains greatly unresolved.

**Results:**

Two new methods, AmpMCF and ShotMCF, for reconstruction of the whole-genome intra-host viral variants and estimation of their frequencies were developed, based on Multicommodity Flows (MCFs). AmpMCF was designed for NGS reads obtained from individual PCR amplicons and ShotMCF for NGS shotgun reads. While AmpMCF, based on covering formulation, identifies a minimal set of quasispecies explaining all observed reads, ShotMCS, based on packing formulation, engages the maximal number of reads to generate the most probable set of quasispecies. Both methods were evaluated on simulated data in comparison to Maximum Bandwidth and ViSpA, previously developed state-of-the-art algorithms for estimating quasispecies spectra from the NGS amplicon and shotgun reads, respectively. Both algorithms were accurate in estimation of quasispecies frequencies, especially from large datasets.

**Conclusions:**

The problem of viral population reconstruction from amplicon or shotgun NGS reads was solved using the MCF formulation. The two methods, ShotMCF and AmpMCF, developed here afford accurate reconstruction of the structure of intra-host viral population from NGS reads. The implementations of the algorithms are available at http://alan.cs.gsu.edu/vira.html (AmpMCF) and http://alan.cs.gsu.edu/NGS/?q=content/shotmcf (ShotMCF).

## Background

RNA-dependent RNA-polymerases of RNA viruses are error prone and frequently generate mutations, accumulation of which results in a diverse intra-host viral population of closely related genetic variants [1,2], commonly termed quasispecies by virologists.

The advent of Next-Generation Sequencing (NGS) presented invaluable opportunity for the in-depth evaluation of viral quasispecies and understanding the structure of viral intra-host populations in unprecedented detail [3,4]. The application of NGS to clinical and public health settings offers prospects for significant improvement in controlling drug resistance [5] and development of novel therapeutics and vaccines [6]. One of the major advantages of NGS is in generating sequence data at a scale that allows not only analysis of intra-host viral variants from a single amplicon or recovery of the consensus full-length genomic sequence but also reconstruction of the population of full-genome quasispecies from an infected host.

The problem of reconstruction of a structure of viral population formulated as *quasispecies spectrum reconstruction problem *was recently addressed in several studies [7-11]. Given a collection of the shotgun or amplicon NGS reads generated from a sample of the viral population, the algorithms reconstruct a set of quasispecies and their relative frequencies. All published algorithms are based on generating graphs of read overlaps and use minimum-cost flows, probabilistic methods, shortest paths, or maximum bandwidth to reconstruct a set of quasispecies from the graphs [7-11]. The accuracy of reconstruction is affected by the heterogeneity of intra-host viral population. The abundance of conserved genomic regions that extend beyond the maximal read length significantly restricts the full-genome quasispecies assembly. Indeed, even short conserved regions at the overlaps of reads significantly increase ambiguity of quasispecies reconstruction.

Most algorithms for the quasispecies spectrum reconstruction implicitly assume that sequence data were obtained using a shotgun experiment. Although the shotgun method is frequently used for reconstruction of long sequences and produces less distortion in frequency of quasispecies than the amplicon-based approach, the available NGS error correction algorithms are most efficient when applied to amplicon-based data [12,13]. Additionally, although most quasispecies spectrum reconstruction algorithms are technically applicable to both types of data, the amplicon-based approaches allow for a greater control over the distribution of reads across the entire sequence of interest, resulting in a more accurate estimation of the structure of viral population [9,10].

In this paper, we consider two methods, AmpMCF and ShotMCF, for reconstruction of the genetic structure of intra-host viral population using either amplicon or shotgun NGS reads, respectively. Both methods are based on the application of MultiCommodity Flow problem (MCF) [14].

## Methods

MCF is a classical optimization problem that searches for *k *flows for *k *source-sink pairs (*s_i_, t_i_*) in a network *N *in order to either minimize the total cost of flows or maximize the total flow subject to capacity and demand constraints.

Quasispecies reconstruction can be formulated as an optimization problem in two ways: (1) identification of the most probable set of quasispecies formed by the largest subset of reads from the data, referred to as packing formulation; and (2) identification of a minimal set of quasispecies explaining all observed reads, referred to as covering formulation. These two formulations, when applied to MCFs, were developed into path packing and path covering algorithms of ShotMCF and AmpMCF, respectively.

### AmpMCF algorithm

We consider an amplicon *A *as a multiset of reads such that each read *r*∈*A *has the same predefined starting and ending position in the genome start(*A*) and end(*A*), respectively. Two amplicons *A_1_*, *A_2 _*are considered overlapped if and only if start(A_1_) ≤ start(*A_2_*) < end(*A_1_*) ≤ end(A_2_). A set of amplicons *A *= {*A*_1_, ..., *A_m_*} is said to be overlapping if and only if *A_i _*and *A*_*i+*1 _overlap for *i *= 1...*m*-1. Given an overlapping set A, we define a partial order < on the set of reads *R *= *A*_1_⋃...⋃*A_m _*as follows: *r *<*r' *if and only if *r*∈*A_i_*, *r'*∈*A*_*i*+1 _and *r *and *r*' are consistent over their overlap of length l_i,i+1 _= end(A_i_)- start(A_i+1_)+1, i.e., the suffix of length l_i,i+1 _of r coincides with the prefix of length l_i,i+1 _of r'.

Given an overlapping set *A *= {*A*_1_, ..., *A_m_*}, we construct an (*m*+2)-staged directed vertex-weighted read-graph as follows: *G *= (*V(G) = V*_1 _⋃ ... ⋃ *V_m _*⋃ {*s, t*}, *E(G)*, *c*), where each *v*∈ *V_i_*, 1 ≤ *i *≤ *m *corresponds to a distinct read *r_v _*∈*A_i_*. An edge (*u*, *v*) ∈ *E(G) *if and only if either *r_u _*<*r_v _*or *u = s*, *v*∈ *A*_1 _or *u*∈*A_m_*, *v *= *t*. We also define the function *c*: *V*_1 _⋃ ... ⋃ *V_m _*→ [0,1], where *c*(*v*) denotes the frequency of the read represented by *v *∈ *V_i _*in amplicon *A_i_*. It is evident that every full-size quasispecies that has a sequenced read from each amplicon *A_i _*corresponds to an (*s*, *t*)-path in the graph *G*.

A bipartite clique of *G *is defined as a set of vertices *C*⊆*V*(*G*) such that *C*⊆*V_i_*⋃*V*_*i+*1 _for some *i *and every vertex from the set *C*⋂*V_i _*is adjacent to every vertex from the set *C*⋂*V*_*i*+1_.

**Lemma 1**. *Consistent overlaps in amplicons A_i_, A_i+1 _correspond to disjoint bipartite cliques in G*.

**Proof**. Suppose the contrary; then there exist vertices *v*, *v*' ∈ *A_i _*and *u*, *u*' ∈*A*_*i*+1_, such that *r_v _*<*r_u_*, *r_v _*<*r*_*u*'_, *r_v' _*<*r_u_*, but it is not true that *r_v' _*<*r*_*u*'_. Since *r*_*v*' _and *r_u _*are comparable but *r*_*v*' _and *r*_*u*' _are not, the prefixes of length *l*_*i,i+*1 _of *r_u _*and *r*_*u*' _must not be consistent. This implies a contradiction with *r_v _*<*r_u _*and *r_v _*<*r*_*u*'_.

**Figure 1:**
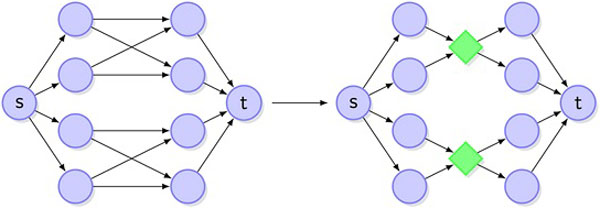
**Bipartite cliques in the read graph are replaced by forks**.

Using this simple finding, we transform the read graph *G *into a new "forked" edge-weighted directed read-graph *H *= (*V(H), E(H), d*) as follows. Consider each *p *× *q*-bipartite clique *C *= *K*_*p,q *_of *G *not containing vertices *s,t*. *C *⊆ *A_i_*⋃*A*_*i*+1 _for some *i*∈{1, ..., *m*-1}. Add a new "fork" vertex *v_fork_*, delete all edges of the bipartite clique *C *and add edges from the sets {(*u,v_fork_*): *u*∈*C*⋂*A_i_*} and {(*v_fork_*, *v*): *v*∈*C*⋂*A*_*i*+1_}. Define a new edge weight function *d *: *E(H) *→ *N *as follows: *d*(*uv_fork_*) = *c(u)*, *d(v_fork_v) = c(v), d(su) = d(vt) *= 0. Figure 1 illustrates this transformation.

As for *G*, every full-size quasispecies corresponds to (*s,t*)-path in the forked read graph *H*. However, *H *is (2*m*+1)-staged directed graph with much fewer edges than *G*: for every bipartite clique *K_p,q _pq *edges in *G *are replaced by only *p+q *edges in *H*. Since in network flow problems variables usually are associated with edges, this reduction is highly useful for the construction of the fast network flows-based algorithm for the quasispecies spectrum reconstruction problem.

The quasispecies reconstruction problem may be restated as the following covering problem:

**Problem 1**. Given a forked read graph *H*, cover *H *with a set of unique (*s,t*)-paths *P_i _*with frequencies *g_i _*such that the total frequency of paths is minimal and for every directed edge (*u,v*)∈ *E(H) *the sum of frequencies of paths containing (*u*, *v*) is at least *d(uv)*.

We next reformulate Problem 1 as an MCF problem. Suppose that *k *is an upper bound for the number of quasispecies (k is the parameter of the algorithm analogous to the parameters of clustering algorithms such as k-means). Then an exact solution of Problem 1 could be obtained using the following Mixed Integer Linear Programming formulation:

(1) $$minimize\underset{\begin{array}{c} i=1,\ldots k\\ \left(s,u\right)\in E\left(H\right) \end{array}}{\sum }g_{su}^{i}$$

s.t.

(2) $$\overset{k}{\underset{i=1}{\sum }}g_{uv}^{i}\geq d\left(uv\right),\;\;\left(u,v\right)\in E\left(H\right)$$

(3) $$\underset{\left(u,v\right)\in E\left(H\right)}{\sum }g_{uv}^{i}=\underset{\left(v,w\right)\in E\left(H\right)}{\sum }g_{vw}^{i},v\in V\left(H\right)\backslash \left\{s,t\right\},\;i=1,\ldots ,k$$

(4) $$\underset{\left(v,u\right)\in E\left(H\right)}{\sum }f_{vu}^{i}\leq 1,v\in V\left(H\right),\;i=1,\ldots ,k$$

(5) $$f_{uv}^{i}\geq g_{uv}^{i},\left(u,v\right)E\left(H\right),\;i=1,\ldots ,k$$

(6) $$f_{uv}^{i}\in \left\{0,1\right\},\;\left(u,v\right)\in E\left(H\right),\;i=1,\ldots ,k$$

(7) $$g_{uv}^{i}\in \left[0,1\right],\left(u,v\right)\in E\left(H\right),\;i=1,\ldots ,k$$

The variables $g_{uv}^{i}$ represent the values of the flow *i *on the edge (*u,v*). With each flow *g^i ^*we associate a binary vectors *f^i ^*such that for every (*u,v*)∈*E(H)*

(8) $$\text{if }g_{uv}^{i}>0\text{ then }f_{uv}^{i}=1$$

This condition is guaranteed by the constraints (5). Constraints (2) and (3) are covering and flow conservation constraints, respectively. Constraints (4) guarantee that flows *g^i ^*are unsplittable for every *i *= 1, ..., *k*, i.e. the edges carrying each flow form a simple (*s,t*)- path *P_i _*in the forked read-graph *H*. In particular, the constraints imply that for every *i *= 1, ..., *k *the values $g_{uv}^{i}$ are equal for all edges of *P_i_*. Therefore $g_{uv}^{i}$ can be interpreted as values proportional to frequencies of quasispecies *i*.

The frequency of *i-*th quasispecies is calculated as the normalized size of the *i*-th flow by the formula

(9) $$\frac{\sum_{su\in E\left(H\right)}g_{su}^{i}}{\sum_{i=1}^{k}\sum_{su\in E}g_{su}^{i}}.$$

### ShotMCF algorithm

The input is a set of distinct reads *R *with counts (*c_v _*: *v*∈ *R*) and a set of candidate sequences *Q *= {*q*_1_, ..., *q_k_*} generated using the max bandwidth method of ViSpA. We construct the directed read graph *G *= (*V, E*) as follows:

1) for each read *r_v _*∈*R *aligned with the reference sequence add a vertex *v*∈*V*; the consensus of candidate sequences can be used as a reference;

2) the directed edge (*u, v*) belongs to *E *if and only if some suffix of *r_u _*overlaps with a prefix of *r_v _*and the two reads agree inside the overlap;

3) for each candidate sequence *q_i_*∈ *Q *add a source *s_i _*and a sink *t_i_*. Add edges (*s_i_,v*) and (*v,t_i_*) for each vertex *v*∈*R *such that *r_v _*coincides with the prefix or suffix of *q_i_*, respectively.

Let a read *r_v _*of length *l *be aligned with a candidate sequence *q_i _*and its alignment have *j *mismatches (replacements, insertions and deletions). Let *p^i^_v _*be the probability that read *r_v _*was obtained from the sequence *q_i_*. This probability can be estimated as

(10) $$p_{v}^{i}={\left(\frac{\varepsilon }{3}\right)}^{j}{\left(1-\varepsilon \right)}^{l-j},$$

where *ε *is the sequencing error rate, i.e. the probability of error per nucleotide. Note that the analogous formula was used in the quasispecies theory for the calculation of the probability of mutation between two different quasispecies [15].

Using the read-graph constructed above, the quasispecies frequencies estimation problem can be formulated in terms of MCF as follows. Each (*s_i_*, *t_i_*)-path corresponds to some full-genome quasispecies, which can coincide with *q_i _*with a probability depending on values *p^i^_v_*. By using *p^i^_v _*as coefficients in the MCF objective function, we arrive to the following formulation:

(11) $$maximize\underset{v\in V}{\sum }\overset{k}{\underset{i=1}{\sum }}p_{v}^{i}g_{v}^{i}$$

s.t.

(12) $$\overset{k}{\underset{i=1}{\sum }}g_{v}^{i}\leq c_{v},v\in V$$

(13) $$\underset{uv\in E}{\sum }g_{uv}^{i}=\underset{vw\in E}{\sum }g_{vw}^{i},\;v\in V\backslash \left\{s_{i},t_{i}\right\},\;i=1,\ldots ,k$$

(14) $$g_{uv}^{i}\geq 0,\;uv\in E,i=1,\ldots ,k$$

Here $g_{uv}^{i}$ are flow variables. $g_{v}^{i}=\sum_{uv\in E}g_{uv}^{i}$ are auxiliary variables used for the simplicity of notations, which represent total flow through vertices *v*∈*V*. The resulted flow is fractional and can split, thus allowing for read errors and mutations. (11)-(14) is a variant of MCF, where vertex capacity constraints are used instead of edge capacity constraints. Once the problem is solved, the frequency of each candidate quasispecies could be estimated using (9).

## Results

In order to validate the devised methods, we used reads simulated from experimentally identified intra-host HCV variants or quasispecies.

The simulated reads were generated using individual 1734-nt sequences derived from the E1/E2 genomic region of intra-host HCV variants [16]. For each run of the algorithm, quasispecies populations were generated using 10 randomly selected sequences with randomly assigned frequencies. Quasispecies frequencies were generated according to uniform, geometric, and skewed distributions.

1) In the uniform distribution all sequences have approximately equal frequencies, which were calculated as normalized numbers of times each sequence was chosen in 1000 independent trials, where at each trial one of sequences was randomly chosen with an equal probability.

2) In the geometric distribution frequencies form a geometric progression. The frequencies were calculated by taking 10 first terms in geometric progressions and normalizing them.

3) In the skewed distribution one of the sequences has a high frequency, while the remaining sequences have uniformly low frequencies (generated as in 1).

The read lengths followed a normal distribution with mean value of 320nt and variance of 10nt. The number of reads in each simulated data set varied from 5K to 300K for ShotMCF and from 5K to 100K for AmpMCF. Shotgun reads were simulated using FlowSim [17]. We generated amplicons with the length of 320nt and difference of 250nt between starting positions of consecutive amplicons. The starting position of each amplicon read was chosen among amplicons starting positions using a uniform distribution.

For each size of a data set and for each distribution type 11 independent simulated data sets were generated, averages of measures of algorithms quality were calculated and the statistical significance of algorithms comparison was estimated using a Kruskal-Wallis test [18].

Problems formulations (1)-(7) and (11)-(14) were solved using the IBM ILOG CPLEX solver 12.2 (http://www.ibm.com/software/integration/optimization/cplex-optimizer/) with the default parameters. ILP for AmpMCF was solved in parallel on 16x Intel(R) Xeon(R) CPU X5550 2.67 GHz, 48 GB Memory with a running time limit 5 minutes per problem. LP for ShotMCF was solved in parallel on 24x Intel(R) Xeon(R) CPU E7450 2.40 GHz, 128 GB Memory to optimality. The average running time for solving LP formulation for ShotMCF varied from 30.945 seconds with a standard deviation 11.332 seconds for 5K reads to 352.301 seconds with a standard deviation 56.861 seconds for 300K reads. The average running time for solving ILP formulation for AmpMCF varied from 110.219 seconds with a standard deviation 106.342 seconds for 5K reads to 126.270 seconds with a standard deviation 99.500 seconds for 100K reads.

P-values for a Kruskal-Wallis test were calculated using MATLAB (http://www.mathworks.com/products/matlab/).

### ShotMCF algorithm

The reconstructions obtained using ShotMCF and EM algorithms from ViSpA [8] were compared. It was shown in [8] that ViSpA with EM outperforms state-of-the-art algorithm SHORAH proposed in [7]. Since EM and ShotMCF use the same method for candidate quasispecies generation, both algorithms were evaluated for the accuracy of finding the distribution of quasispecies frequencies. Following [8] and [19], we used two measures of accuracy: Root Mean Square Error (RMSE) and Kullback-Leibler divergence (KLD) [20] between the estimated distribution and the true distribution. KLD is a quasi-metric that measures the distance between two probability distributions *P*=(*p*_1_, ..., *p_n_*) and *W*=(*w*_1_, ..., *w_n_*) by the following formula:

$$KLD\left(P,W\right)=\overset{n}{\underset{i=1}{\sum }}\text{ln}\left(\frac{p_{i}}{w_{i}}\right)p_{i}$$

**Figure 2:**
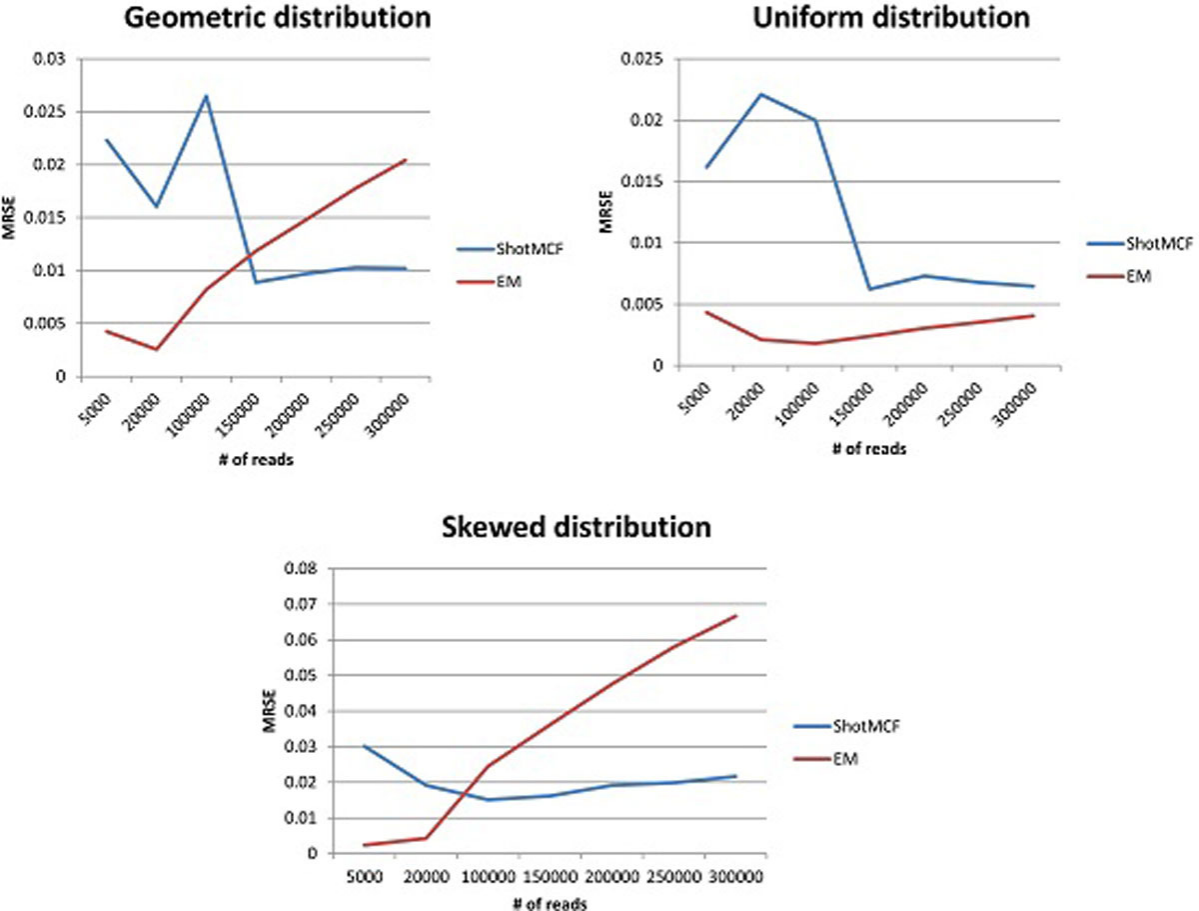
**Comparison of ShotMCF and EM - RMSE**.

**Figure 3:**
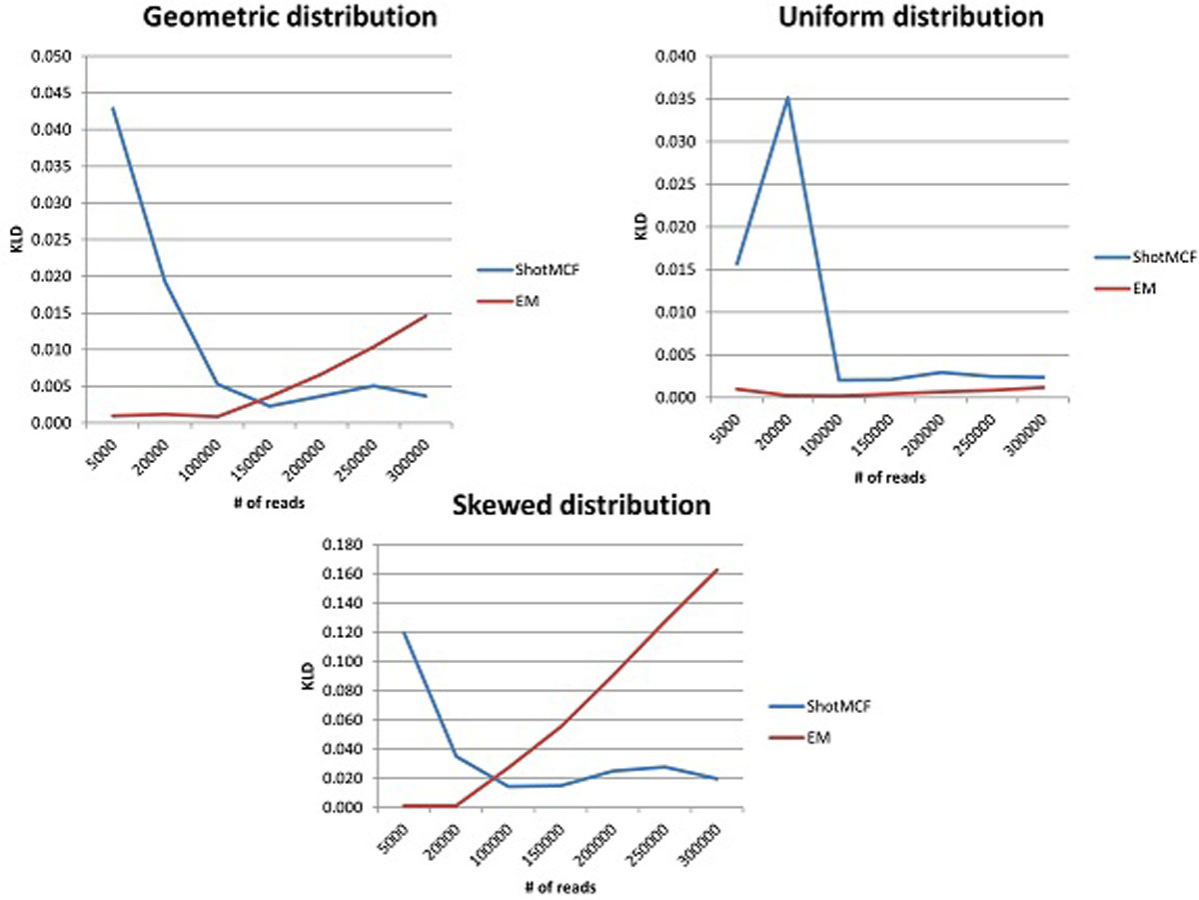
**Comparison of ShotMCF and EM - KLD**.

Figures 2, 3 illustrates the comparison of ShotMCF and EM algorithms. The difference in performance between two algorithms is statistically significant for all distributions and sizes of data. The p-values of a Kruskal-Wallis test are summarized in Table 1.

**Table 1:** Statistical significance of the comparison of ShotMCF and EM

	Geometric distribution						
**# of reads**	5000	20000	100000	150000	200000	250000	300000
**p-value RMSE**	0.000071	0.000071	0.000071	0.002263	0.000122	0.000160	0.000093
**p-value KLD**	0.000071	0.000913	0.000071	0.038598	0.006428	0.016540	0.000071

	**Uniform distribution**						

**# of reads**	5000	20000	100000	150000	200000	250000	300000
**p-value RMSE**	0.000071	0.000071	0.000071	0.000443	0.000566	0.001449	0.005258
**p-value KLD**	0.000071	0.000071	0.000122	0.000345	0.000566	0.001449	0.005258

	**Skewed distribution**						

**# of reads**	5000	20000	100000	150000	200000	250000	300000
**p-value RMSE**	0.000071	0.000071	0.027823	0.000071	0.000720	0.000093	0.000071
**p-value KLD**	0.000071	0.000071	0.027823	0.000071	0.001152	0.001152	0.000071

ShotMCF statistically significantly outperforms EM on large data sets with geometric and skewed distributions, while the quality of EM is higher on small data sets. The quality of quasispecies reconstruction by EM, as implemented in ViSpA [8], declined with the increase in the dataset size for large numbers of reads, and was not significantly affected for ShotMCF. EM produced more accurate results on data sets with up to 300K reads generated using the uniform distribution. However, the trend of decrease in quality of EM estimations suggests that ShotMCF is more accurate on the larger data sets generated using the uniform distribution.

**Figure 4:**
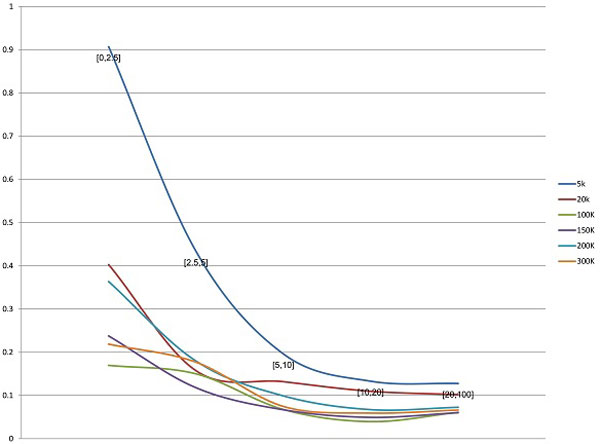
**Dependence between relative error in frequency estimation and an abundance of a variant - ShotMCF**.

The accuracy of frequency estimation for variants with different abundances was analysed (Figure 4). All analysed sequences were partitioned into 5 groups according to their frequencies *f*: *f *≤ 0.025, 0.025 <*f *≤ 0.05, 0.05 <*f *≤ 0.1, 0.1 <*f *≤ 0.2 and *f *> 0.2. x-axis represents the groups and y-axis represents the average relative error of ShotMCF for each group. Frequencies of high-abundance variants were estimated more accurately. The accuracy of frequencies estimation increases monotonically with the abundance and stabilizes approximately at the abundance 0.1. The quality of frequency estimation increases, in general, with the number of reads in data set for all groups.

### AmpMCF algorithm

The reconstructions obtained using AmpMCF (k = 12) and the Maximum Bandwidth (MB) algorithm proposed in [9] were compared. Maximum bandwidth is based on the packing formulation of the quasispecies spectrum reconstruction problem, and was shown to outperform the algorithm for quasispecies spectrum reconstruction from amplicon reads proposed in [10]. The following measures of quality of a solution were used:

1) RMSE

2) Jensen-Shannon divergence (JSD). It replaces KLD used for ShotMCF testing, since for AmpMCF and MB sizes of the reconstructed quasispecies populations may differ from the size of the correct population. JSD differs from KLD divergence due to the addition of a midpoint and is defined as follows:

$$JSD\left(P,W\right)=\frac{1}{2}KLD\left(P,M\right)+\frac{1}{2}KLD\left(W,M\right),$$

where *P *and *S *are probability distributions and $M=\frac{1}{2}\left(P+W\right)$.

3) Sensitivity S, which is defined as

$$S=\frac{\left|TruePositives\right|}{\left|TruePositives\right|+|FalseNegatives|}$$

4) Positive predicted value (PPV), which is defined as

$$PPV=\frac{\left|TruePositives\right|}{\left|TruePositives\right|+|FalsePositives|}$$

Here, if CandQ is the set of quasispecies found by the algorithm and SimQ is the set of simulated quasispecies, then TruePositives = CandQ⋂SimQ, FalseNegatives = SimQ\CandQ and FalsePositives = CandQ\SimQ.

RMSE and JSD measure the quality of quasispecies frequencies estimation, and Sensitivity and PPV measure the quality of assembled quasispecies. Sensitivity is a measure of the positive identifications, which is defined as the percentage of correctly assembled quasispecies out of the population. PPV is a measure of the negative identification, which is defined as the percentage of correctly identified quasispecies over all assembled quasispecies.

**Figure 5:**
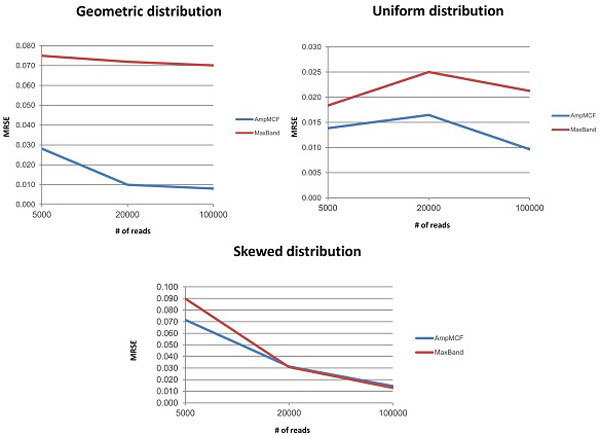
**Comparison of AmpMCF and Maximum Bandwidth - RMSE**.

**Figure 6:**
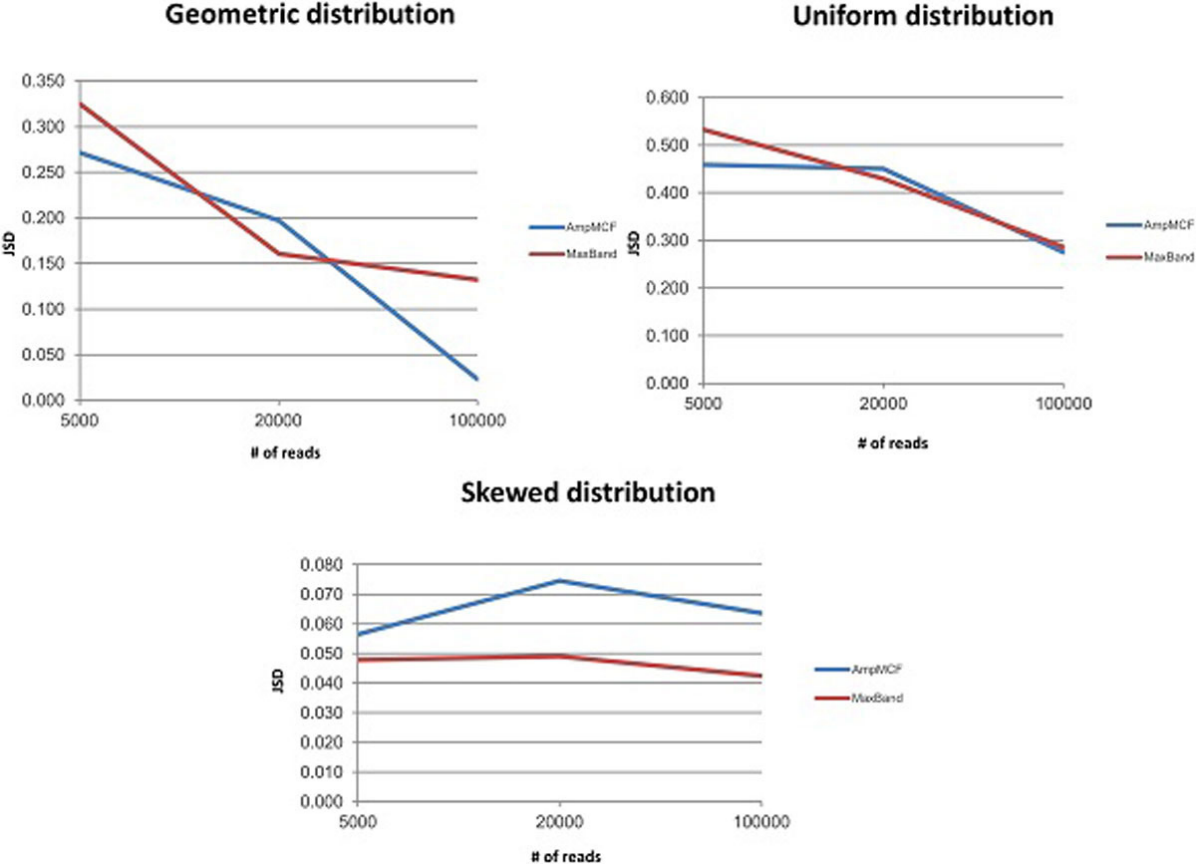
**Comparison of AmpMCF and Maximum Bandwidth - JSD**.

**Figure 7:**
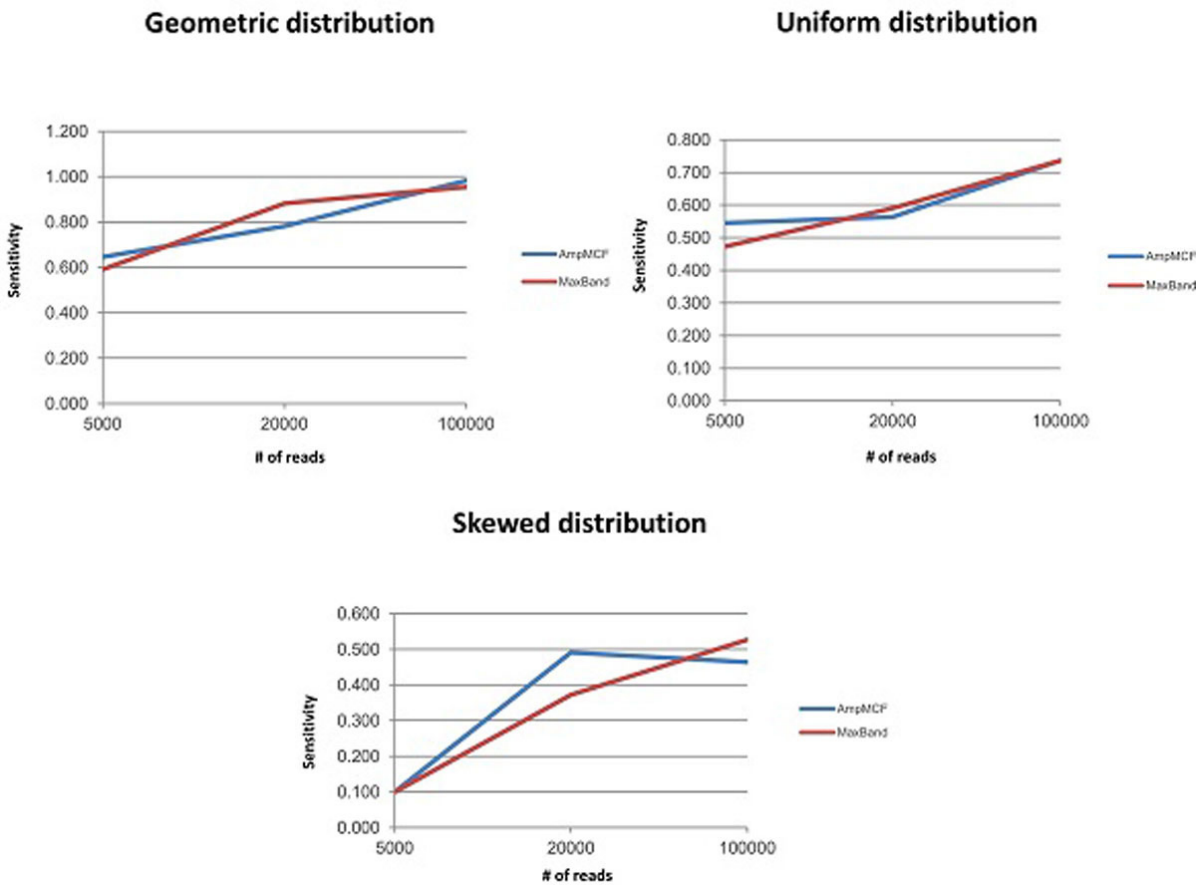
**Comparison of AmpMCF and Maximum Bandwidth - Sensitivity**.

**Figure 8:**
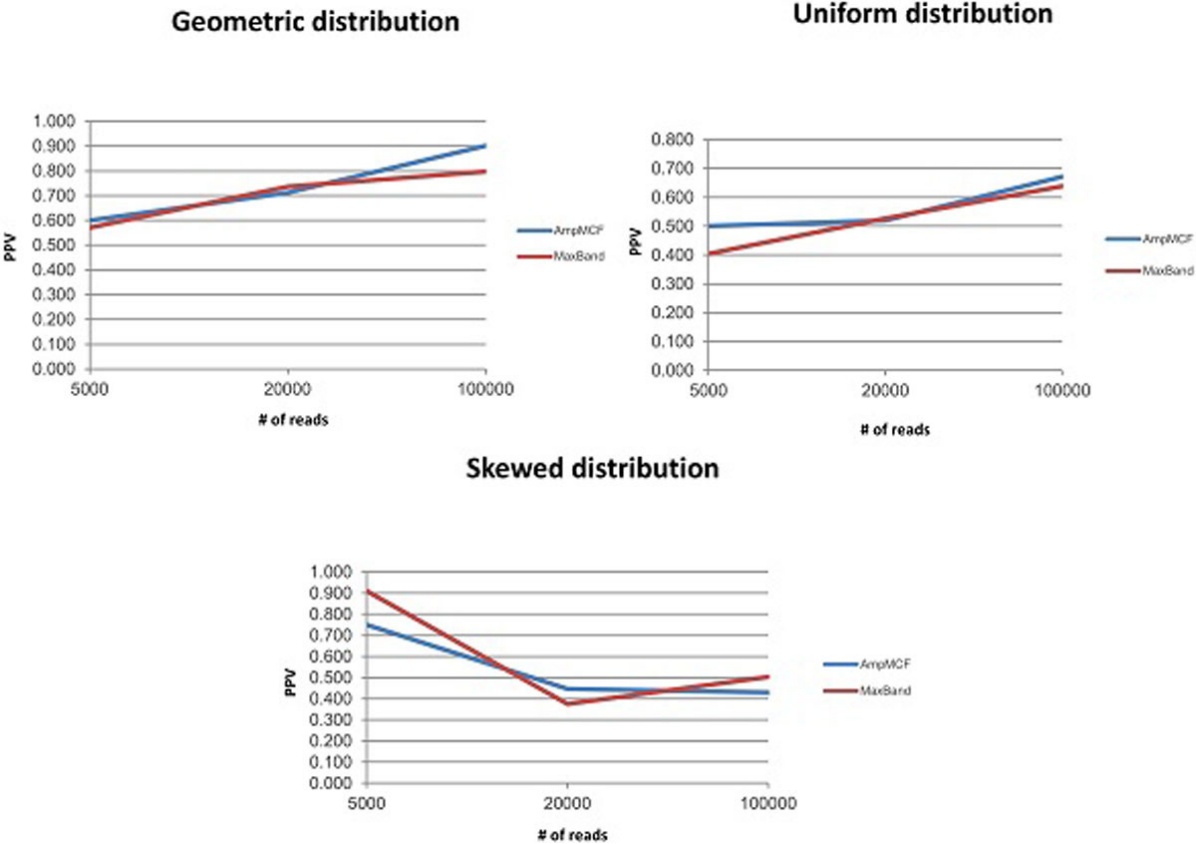
**Comparison of AmpMCF and Maximum Bandwidth - PPV**.

Figures 5, 6, 7, 8 illustrate the comparison of AmpMCF and Maximum Bandwidth algorithms, and Table 2 summarizes statistical significance of the comparison of the algorithms.

**Table 2:** Statistical significance of the comparison of AmpMCF and EM

	Geometric distribution		
**# of reads**	5000	20000	100000
**p-value RMSE**	0.001100	0.000069	0.000070
**p-value JSD**	0.200130	0.742240	0.000718
**p-value S**	0.46294	0.11743	0.84517
**p-value PPV**	0.66827	0.79078	0.037853

	**Uniform distribution**		

**# of reads**	5000	20000	100000
**p-value RMSE**	0.122800	0.061063	0.015030
**p-value JSD**	0.339790	0.818120	0.742170
**p-value S**	0.34978	0.78918	0.89135
**p-value PPV**	0.13832	0.89501	0.50755

	**Skewed distribution**		

**# of reads**	5000	20000	100000
**p-value RMSE**	0.469220	0.717980	0.224440
**p-value JSD**	0.211260	0.004284	0.023486
**p-value S**	-	0.12341	0.39881
**p-value PPV**	0.20846	0.53018	0.40896

According to RMSE, AmpMCF statistically significantly outperforms Maximum Bandwidth for all sizes of data sets with the geometric distributions, and for large data sets with the uniform distribution. Although AmpMCF exceeded in accuracy Maximum Bandwidth on the 5K and 20K datasets with the uniform distribution, the difference in performance was statistically insignificant, with p-value being slightly greater than the statistical significance threshold of 5%. For the skewed distribution the results were comparable without statistically significant advantage of one algorithm over the other.

According to JSD and PPV, ShotMCF statistically significantly outperforms Maximum Bandwidth on the 100K data sets with the geometric distribution, while Maximum Bandwidth had the lower JSD values on the 20K and 100K data sets with the skewed distribution. For all other measures, sizes and distributions the results were comparable with no statistically significant advantage of one algorithm over the other. The p-value for S could not be calculated for the 5K data sets with the skewed distribution, since both algorithms were equally sensitive on all test examples.

So AmpMCF outperformed Maximum Bandwidth in quasispecies frequencies estimation for populations with geometric and uniform distributions, while both algorithms showed a similar performance in quasispecies sequence reconstruction.

**Figure 9:**
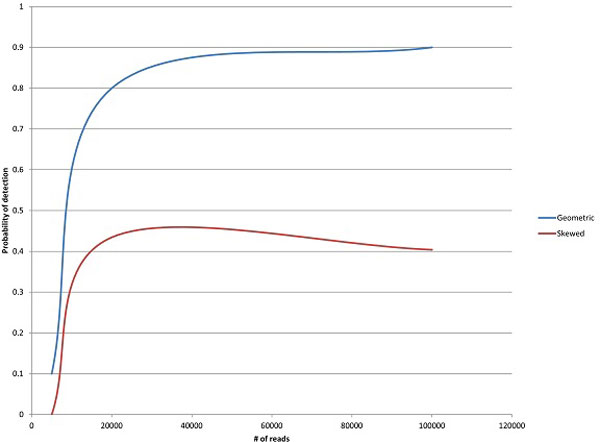
**Probabilities of detection of low-frequency variants (< 0.025) for the geometric and skewed distributions - AmpMCF**.

The low sensitivity of AmpMCF and Maximum Bandwidth on the 5K data set with the skewed distribution is associated with the erroneous reconstruction of low-abundance variants by both algorithms, with only a dominant variant being correctly identified. For larger data sets, populations with the skewed distributions were reconstructed much more successfully and variants with frequencies as low as 0.8% were detected. It should be also noted that low-frequency variants were detected with higher probability in populations with the geometric distribution (Figure 9). It suggests that the recoverability of low-frequency variants depends on the structure of a population and that the coverage provided by data sets of 5K reads is insufficient for low-frequency variants detection, if the population contains a dominant high-frequency variant.

**Figure 10:**
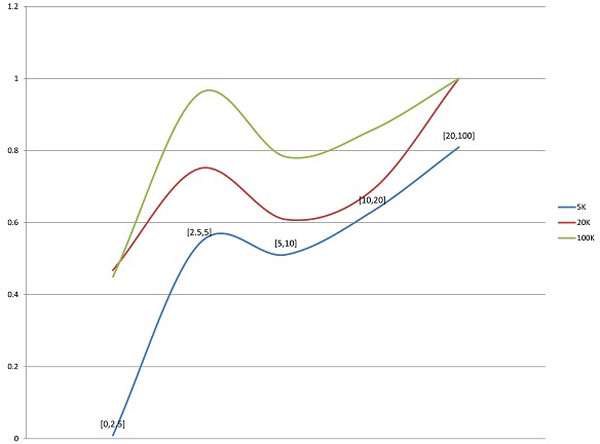
**Probabilities of detection of quasispecies depending on their frequencies - AmpMCF**.

In general, abundances of variants greatly affect their recoverability, with high-frequency variants being easier to detect (Figure 10). As above, all analysed sequences in Figure 10 were partitioned into 5 groups according to their frequencies f: f ≤ 0.025, 0.025 < f ≤ 0.05, 0.05 < f ≤ 0.1, 0.1 < f ≤ 0.2 and f > 0.2. x-axis represents the groups and y-axis the probability of variant recovery in each group. The probabilities of detection of variants within each group increase with the number of reads in a data set. While the probability of reconstruction of a variant with frequency less than 2.5% from the 5K data set was only 0.0092, all variants with frequencies greater than 20% were reconstructed from 20K and 100K data sets.

**Figure 11:**
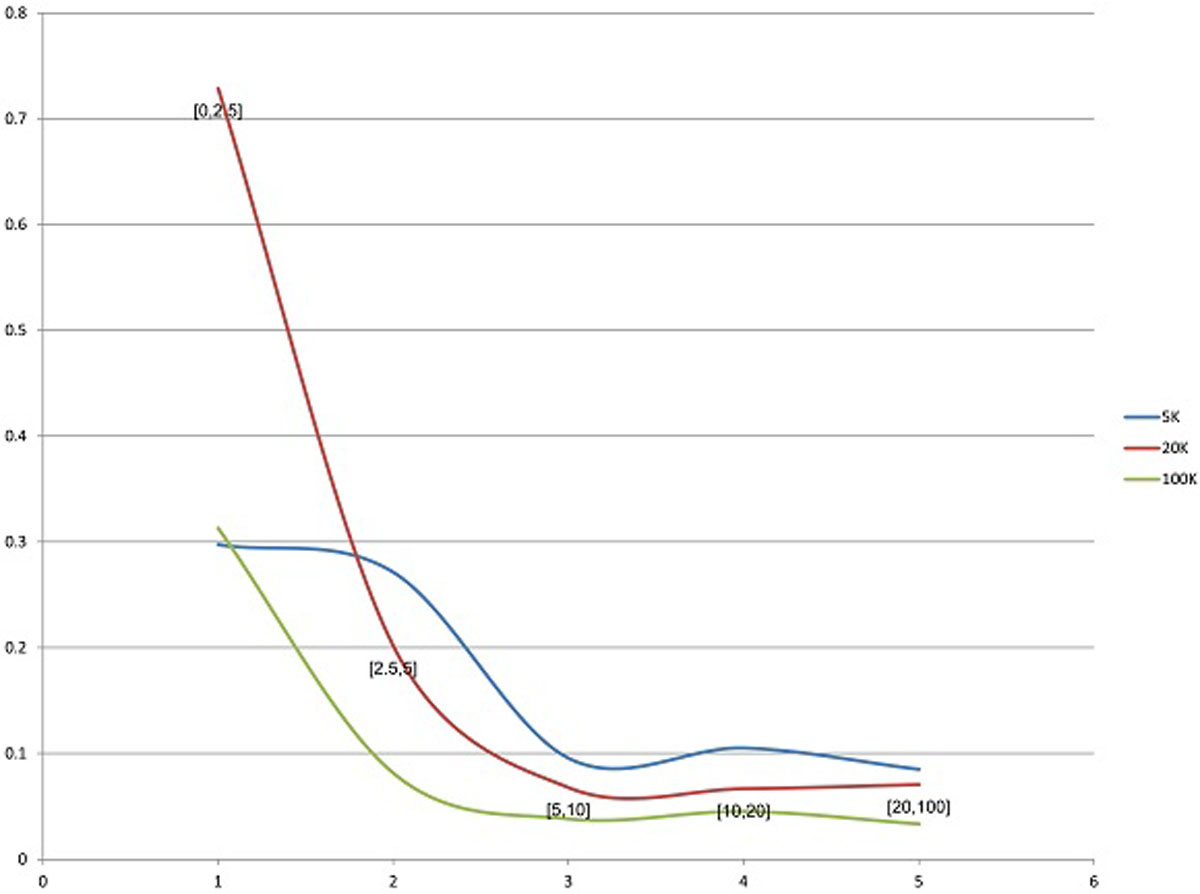
**Dependence between relative error in frequency estimation and an abundance of a variant - AmpMCF**.

The accuracy of frequency estimation for detected variants with different abundances is illustrated by Figure 11. As for ShotMCF, the accuracy of frequency estimation increases with the abundance and stabilizes approximately at the abundance 0.1. In general, the accuracy of frequency estimation increases with the number of reads in a data set for all groups except for the group of low-frequency variants. The small value of RE for low-frequency variants from the 5K data sets can be explained with a low detection rate of such variants, which renders their RE undefined.

## Discussion

Two different network-flows based formulations applicable to quasispecies frequency reconstruction problem were developed. The first quasispecies spectrum reconstruction method based on network flows (NF) was proposed in [11]. However, usage of NF in that method does not allow the direct reconstruction of quasispecies sequences and their frequencies. Rather, it selects pairs of overlapping reads that belong to the same sequence variant. For the direct quasispecies spectrum reconstruction the second stage of the algorithm was proposed, which involves finding edge-disjoint paths in the network modified according to the results of the NF stage. The network modification substantially increases the number of edges; therefore the method is computationally expensive.

AmpMCF extends the concept developed in [11]. It replaces NF with MCF, which allows for joining both stages of algorithm from [11] in a single MCF formulation and for solving it using a single algorithm. Such approach is more effective and allows for increasing quality of the solution. Moreover, instead of increasing the size of the network, AmpMCF allows to decrease it, thus making the problem much more computationally tractable.

ShotMCF extends the ViSpA algorithm described in [8]. The method proposed in [8] consists of two stages: generation of candidate quasispecies sequences from shotgun NGS reads using Maximum Bandwidth paths in the read graph and estimation of their frequencies using the Expectation Maximization (EM) algorithm [21]. ShotMCF models and solves the quasispecies frequency estimation problem using MCF instead of EM. Unlike AmpMCF and the algorithm from [11], it is a packing algorithm that invokes the vertex rather than edge capacity constraints and does not require integer variables. This new method in combination with the candidate sequences generation algorithm from [8] presents a novel framework for the reliable reconstruction of quasispecies spectrum.

The formulation for AmpMCF could not be applied to shotgun data since it assumes that each full-length sequence corresponds to a unique (s,t)-path in the read graph. However, this is not true for the shotgun data since certain sequences can be assembled from reads through different paths. This observation taken together with consideration of the structure of the read graph described by Lemma 1 indicates that the formulation is more suitable for amplicons. The analogue of AmpMCF for a shotgun data is the NF-based algorithm from [8]. However, as aforementioned, it is computationally expensive and known to be outperformed by ViSpA.

Although the formulation of ShotMCF is applicable to amplicons, AmpMCF is more suitable for this task since ShotMCF handles only the second stage of quasispecies spectrum reconstruction problem, with the first stage being the candidate sequence generation adopted from ViSpA; while AmpMCF incorporates the whole problem into a single formulation.

The structure of the read graph explains a better match of the amplicon data to the covering rather than to packing formulation implemented by Maximum Bandwidth. According to Lemma 1, consistent overlaps between consecutive amplicons form bipartite cliques in a read graph. Edges within each bipartite clique are equal in respect to choosing (s,t)-paths in a read graph. This leads to a large number of peer alternatives for quasispecies assembling, indicating the need to search for the most parsimonious solution. The NF-based formulation with parsimony as an objective function and without predefined flow sizes requires covering constraints, and, therefore, leads to the covering formulation.

The advantage of ShotMCF method over EM-based method of ViSpA originates from enforcing uniformity of quasispecies coverage and using more accurate formula for the probability of emission of a given read from a given candidate sequence. The major advantage of the EM algorithm over the current version of ShotMCF is a greater speed and reduced requirements for computational resources such as computer memory and number of parallel processors. The reason is that MCF is implemented directly using linear programming formulation. It is expected that application of faster methods; e.g., based on lagrangian relaxations or Bender decomposition, should dramatically increase performance of ShotMCF.

It should be noted that MCF formulations assume absence of gaps in coverage. Although such gaps interrupt the assembly of the entire sequence, the genomic regions covered with reads can be identified using a reference sequence and quasispecies can be estimated with MCF-based algorithms for each region independently.

## Conclusions

Two novel methods were developed for the reconstruction of the structure of viral population from the NGS shotgun and amplicon reads. Both methods are based on MCF and found suitable for the reliable assembly of viral quasispecies and estimation of their frequencies.

## Competing interests

Authors declare that they have no competing interests.

## Authors' contributions

PS developed the algorithms and wrote the manuscript. NM developed, implemented and tested AmpMCF algorithm. AA developed, implemented and tested ShotMCF algorithm. BT prepared the testing data. IM contributed to designing the algorithms and writing the manuscript. YK contributed to designing the algorithms and writing the manuscript. AZ developed the algorithms, wrote the manuscript and supervised the project. All authors read and approved the final manuscript.

## Declarations

Publication of this article was funded intramurally by Centers for Disease Control and Prevention.

This article has been published as part of *BMC Bioinformatics *Volume 14 Supplement 9, 2013: Selected articles from the 8th International Symposium on Bioinformatics Research and Applications (ISBRA'12). The full contents of the supplement are available online at http://www.biomedcentral.com/bmcbioinformatics/supplements/14/S9.

## References

[B1] Domingo E Biological significance of viral quasispecies Viral Hepatitis Rev 2 1996 247 261

[B2] Duarte EA Novella IS Weaver SC Domingo E Wain-Hobson S Clarke DK Moya A Elena SF de la Torre JC Holland JJ RNA Virus Quasispecies: Significance for Viral Disease and Epidemiology Infectious Agents and Disease 1994 3 4 201 214 7827789

[B3] Beerenwinkel N Zagordi O Ultra-deep sequencing for the analysis of viral populations Curr Opin Virol 2011 1 5 413 8 10.1016/j.coviro.2011.07.008 22440844

[B4] Beerenwinkel N Günthard HF Roth V Metzner KJ Challenges and opportunities in estimating viral genetic diversity from next-generation sequencing data Front Microbiol 2012 3 329 22973268 10.3389/fmicb.2012.00329 PMC3438994

[B5] Skums P Campo D Dimitrova Z Vaughan G Lau D Khudyakov Y Numerical detection, measuring and analysis of differential interferon resistance for individual HCV intra-host variants and its influence on the therapy response In Silico Biology 2011 11 1 7 23202427 10.3233/ISB-2012-0460

[B6] Luciani F Bull RA Lloyd AR Next generation deep sequencing and vaccine design: today and tomorrow Trends Biotechnol 2012 30 9 443 52 10.1016/j.tibtech.2012.05.005 22721705 PMC7127335

[B7] Zagordi O Bhattacharya A Eriksson N Beerenwinkel N ShoRAH: estimating the genetic diversity of a mixed sample from next-generation sequencing data BMC Bioinformatics 2011 12 119 21521499 10.1186/1471-2105-12-119 PMC3113935

[B8] Astrovskaya I Zelikovsky A Inferring Viral Quasispecies Spectra from 454 Pyrosequencing Reads BMC Bioinformatics 2011 12 Suppl 6 S1 10.1186/1471-2105-12-S6-S1 21989211 PMC3194189

[B9] Mancuso N Tork B Skums P Mandoiu I Zelikovsky A Viral Quasispecies Reconstruction from Amplicon 454 Pyrosequencing Reads In Silico Biology 2011 11 1 13 23202425 10.3233/ISB-2012-0458

[B10] Prosperi MC Prosperi L Bruselles A Abbate I Rozera G Vincenti D Capobianchi MR Ulivi G Combinatorial Analysis and Algorithms for Quasispecies Reconstruction using Next-Generation Sequencing BMC Bioinformatics 2011 12 5 10.1186/1471-2105-12-5 21208435 PMC3022557

[B11] Westbrooks K Astrovskaya I Campo D Khudyakov Y Berman P Zelikovsky A HCV Quasispecies Assembly using Network Flows Proc International Symposium Bioinformatics Research and Applications 2008 159 170

[B12] Skums Pavel Dimitrova Zoya Campo David S Vaughan Gilberto Rossi Livia Forbi Joseph C Yokosawa Jonny Zelikovsky Alex Khudyakov Yury Efficient error correction for next-generation sequencing of viral amplicons BMC Bioinformatics 2012 13 Suppl 10 S6 10.1186/1471-2105-13-S10-S6 22759430 PMC3382444

[B13] Quince C Removing noise from pyrosequenced amplicons BMC Bioinformatics 2011 12 38 10.1186/1471-2105-12-38 21276213 PMC3045300

[B14] Ahuja RK Magnanti TL Orlin JB Network Flows: Theory, Algorithms, and Applications 1993 Prentice Hall

[B15] Nowak M 2006 Evolutionary dynamics, Belknap Press of Harvard University Press

[B16] Von Hahn T Yoon JC Alter H Rice CM Rehermann B Balfe P Mckeating JA Hepatitis C virus continuously escapes from neutralizing antibody and t-cell responses during chronic infection in vivo Gastroenterology 2007 132 667 678 10.1053/j.gastro.2006.12.008 17258731

[B17] Balser S Malde K Lanzen A Sharma A Jonassen I Characteristics of 454 pyrosequencing data-enabling realistic simulation with FlowSim Bioinformatics 2010 26 i420 5 10.1093/bioinformatics/btq365 20823302 PMC2935434

[B18] Kruskal W Wallis W Use of ranks in one-criterion variance analysis Journal of the American Statistical Association 1952 47 260 583 621 10.1080/01621459.1952.10483441

[B19] Eriksson N Pachter L Mitsuya Y Rhee SY Wang C Viral population estimation using pyrosequencing PLoS Comput Biol 2008 4 e1000074 10.1371/journal.pcbi.1000074 18437230 PMC2323617

[B20] Kullback S Leibler RA On information and sufficiency The Annals of Mathematical Statistics 1951 22 1 79 86 10.1214/aoms/1177729694

[B21] Dempster AP Laird NM Rubin DB Maximum Likelihood from Incomplete Data via the EM Algorithm Journal of the Royal Statistical Society. Series B (Methodological) 1977 39 1 1 38

